# Prevalence, characteristics and risk factors for chronic lower-limb oedema in the older population living in the community or in an aged-care home: a systematic review

**DOI:** 10.1093/ageing/afag277

**Published:** 2026-09-22

**Authors:** Maree O’connor, Suzanne Kuys, Michael Steele, Peter Franks, Helen Badge

**Affiliations:** Melbourne Campus, School of Allied Health, Australian Catholic University, 115 Victoria Parade, Fitzroy, Victoria 3065, Australia; Brisbane Campus, School of Allied Health, Australian Catholic University, 1100 Nudgee Road, Banyo, Queensland 4014, Australia; Brisbane Campus, School of Allied Health, Australian Catholic University, 1100 Nudgee Road, Banyo, Queensland 4014, Australia; International Lymphoedema Framework, London, UK; Faculty of Health Sciences, School of Allied Health, Australian Catholic University, North Sydney, New South Wales, Australia

**Keywords:** lymphoedema, prevalence, edema, leg, ageing, systematic review, older people

## Abstract

**Introduction:**

Chronic lower-limb oedema co-occurs with multimorbidity, functional limitations and increased risk of wounds and cellulitis. Findings vary across settings and methodologies.

**Objectives:**

To synthesise the prevalence, characteristics and risk factors for chronic lower-limb oedema in older adults living in the community or aged-care homes.

**Methods:**

A systematic review was conducted according to PRISMA 2020 guidelines. MEDLINE, EMBASE, Scopus, Web of Science and Cochrane were searched to 25 March 2026. Eligible studies included adults aged ≥60 years in community or aged-care settings and reported prevalence, characteristics or risk factors for chronic lower-limb oedema. Two reviewers independently screened studies, extracted data and assessed methodological quality.

**Results:**

Sixteen studies met inclusion criteria; 14 were conducted in community settings and two in aged-care homes. Community population-based studies estimated prevalence between 22.7% and 26.4%. Aged-care home prevalence ranged from 10.0% to 27.5% but discretionary exclusion of residents may bias estimates. Chronic lower-limb oedema commonly co-occurred with diabetes, heart disease, obesity (reported at high prevalence across studies), cellulitis and wounds. Evidence for independent risk factors was limited. Substantial heterogeneity reflected differences in study populations, sampling strategies and assessment methods limiting generalisability of findings. Methodological quality varied, particularly in population description and outcome measurement.

**Conclusion:**

Chronic lower-limb oedema appears common ranging from 22.7% to 26.4% prevalence in community settings and up to 27.5% in aged-care homes. Despite potential clinical and economic burden, the evidence base, particularly within aged-care homes, remains limited. Screening may be warranted in these high-risk groups.

## Key Points

- Chronic lower-limb oedema affects around 1 in 4 adults ≥60, representing a substantial, under-recognised burden.
- Evidence on chronic lower-limb oedema in aged care homes is limited, leading to underestimation in this high-risk population.
- Chronic lower-limb oedema in older adults often coexists with multimorbidity, especially diabetes, heart disease and obesity.
- Lower limb oedema is frequently associated with complications including cellulitis, wounds and functional limitation.
- Evidence for independent risk factors remains limited due to heterogeneous methods and inconsistent reporting across studies.

## Introduction

Chronic lower-limb oedema in older people is associated with reduced health, quality of life and increased costs. Chronic oedema is considered an umbrella term encompassing lymphoedema (structural causes) and filtration oedema (excess fluid leaking from the bloodstream into tissues such as in heart failure and venous disease) [1, 2]. It is commonly defined as swelling persisting for at least 3 months although the terms chronic oedema, persistent oedema and lymphoedema are often used interchangeably [3–5]. Regardless of aetiology, lower limb oedema reflects disruption of tissue fluid homeostasis arising from an imbalance between microvascular filtration and lymphatic clearance, with the degree of lymphatic dysfunction influencing clinical presentation and tissue changes [6].

Chronic oedema is associated with substantial burdens, including reduced quality of life, poor health, loss of mobility and independence, and substantial personal and financial costs. Impaired mobility and increased susceptibility to cellulitis and wounds have been linked to chronic lower-limb oedema [7–11]. Chronic lower-limb oedema commonly co-occurs with age-related comorbidities, including cardiovascular disease, stroke, vascular disorders and diabetes [12]. Complications of chronic oedema include recurrent cellulitis, wounds, reduced mobility and independence [7, 13]. These can contribute to substantial economic and healthcare costs [11] beyond those attributable to the co-occurring comorbidities. For example, a hospitalised cellulitis episode in patients with lower-limb chronic oedema has been estimated to cost AUD $13 567 [11].

Despite the high burden, chronic lower-limb oedema is a potentially manageable condition. Early identification enables early intervention, which may reduce oedema-related complications [7, 14, 15]. However, the prevalence of chronic oedema in older people is unclear. Research reports varying prevalence estimates ranging from 2.6% in an outpatient setting [16], 24% [17] in a community setting and up to 54% [17] in aged-care homes. Given that there are an estimated 1.1 billion adults aged ≥60 years globally, rising to 1.4 billion by 2030 [18], prevalence should be explored separately for population-based studies, non-population-based outpatient/specialist services and aged-care homes, as each setting represents a distinct population.

Previous systematic reviews examining the prevalence of lower-limb oedema have primarily focused on specific disease populations, such as individuals with cancer [19] or filariasis [20], with older adults included as part of broader study cohorts. Existing studies are methodologically heterogeneous limiting comparability, and evidence from aged-care home settings remains sparse, constraining understanding in this high-risk population. Studies have used varying assessment tools, employing clinical [9, 16, 21–23] or self-report methods [12]. Additionally, some investigations have included both upper- and lower-limb oedema without disaggregating results by limb type [17, 21].

To date, no systematic review has reported on the prevalence, characteristics and risk factors of chronic lower-limb oedema specifically among older adults living in community and residential aged-care settings. Improved understanding of the burden, clinical presentation and determinants of chronic lower-limb oedema in this population may help targeted screening, monitoring and preventative skin and wound care to older adults most likely to develop oedema and its complications, including cellulitis and leg ulceration. Therefore, the aims of this systematic review were to evaluate the prevalence, characteristics and risk factors of chronic lower-limb oedema in the older population living in the community and in aged-care homes.

## Methods

### Study sources and search

This search adhered to the Preferred Reporting Items for Systematic reviews and Meta-Analyses (PRISMA) 2020 guidelines [24], with prior International prospective register of systematic reviews (PROSPERO) registration (CRD420251051535). Six databases were searched, including Medical Literature Analysis and Retrieval System Online (MEDLINE), Cumulative Index to Nursing and Allied Health Literature (CINAHL), Excerpta Medica Database (EMBASE), Scopus, Web of Science and Cochrane Library. Searches were taken from database inception to 25 March 2026. Search terms included older adult, aged, chronic, oedema, lymphoedema, lower limb and associated synonyms for these words. MEDLINE search strings are provided in Appendix S1 (See Supplementary Data section). These search strings were adapted across the remaining databases.

### Inclusion and exclusion criteria

Eligible studies were published quantitative studies with multiple participants, involving older adults aged ≥60 years living in the community or aged-care homes (also known as residential aged-care, residential facilities nursing homes or homes for the aged). Respite care was also included. Data sources included community clinics, outpatient hospital clinics, individual homes and aged-care homes. Data from outpatient hospital/specialist services were reported as part of the community data. The condition of interest was chronic oedema or lymphoedema of the lower limbs as defined by the study and assessed by clinical evaluation, observation or self-report.

Studies were required to present lower-limb data separately when data from other body regions were also reported. Eligible outcomes included prevalence, characteristics and, where available, risk factors for chronic lower-limb oedema. Studies were excluded if they included only participants <60 years, or if data for those aged ≥60 years were not reported separately. Studies were also excluded if conducted exclusively among hospital inpatients, addressed only acute oedema, or focused solely on upper limb oedema. Experimental intervention studies, including randomised controlled trials, literature and systematic reviews, letters, case reports, conference abstracts and viewpoints were excluded.

### Study selection

Retrieved records were uploaded into Covidence software and duplicates were removed. Records not published in English were translated with Google Translate and then cross-checked by a health professional fluent in the language for translation authenticity. Two independent reviewers screened both title/abstract and full text (M.O., S.K.) for inclusion facilitated by a flow chart. In case of disagreement, consensus was reached on inclusion by discussion and if necessary, a third researcher (H.B.) was consulted. Additional studies were identified through screening reference lists of included articles. Abstracts and full texts of these identified studies were independently screened by two reviewers to determine eligibility for inclusion. Eligibility criteria were refined during preliminary screening to exclude studies exclusively investigating cancer, as these studies constituted a clinically distinct population that was not aligned with the review objectives.

### Data extraction

A purpose-designed Excel spreadsheet was used for data extraction. Data were extracted independently by two reviewers (M.O., S.K.) with discrepancies resolved through discussion. Additional information was sought from study investigators where data were unclear or unavailable in the publication. Data extracted included publication year, country of study, design, aims, recruitment, definition of chronic oedema and oedema assessment method (including, if available, reliability and validity of the assessment method). Participant demographic data extracted included age, gender, care setting, comorbidities, leg oedema, body mass index, wounds, episodes of cellulitis, pressure injuries, mobility level and physical activity. During full-text screening, where studies reported oedema without presenting extractable lower-limb-specific data, authors were contacted for clarification.

### Quality assessment

Risk of bias was assessed using the Joanna Briggs Institute (JBI) Critical Appraisal Checklist [25] for the appropriate study (Table 1). Data were assessed using the checklist independently by two people (M.O., S.K.) with discrepancies resolved through consensus. For each study, the proportion of ‘Yes’ responses was calculated for each question. An overall average proportion of ‘Yes’ responses was calculated across all included studies. In addition, the proportion of ‘Yes’ responses was calculated for each checklist item, where applicable [26, 27]. Thresholds for low (≥70% Yes), moderate (50–69% Yes) and high (<50% Yes) risk were used [27, 28]. Grading of Recommendations Assessment, Development and Evaluation (GRADE) was not applied due to substantial heterogeneity across studies preventing meaningful evaluation of consistency, with prevalence differences likely reflecting true population and setting variation [29].

**Table 1:** JBI critical appraisal for studies reporting prevalence and incidence data.

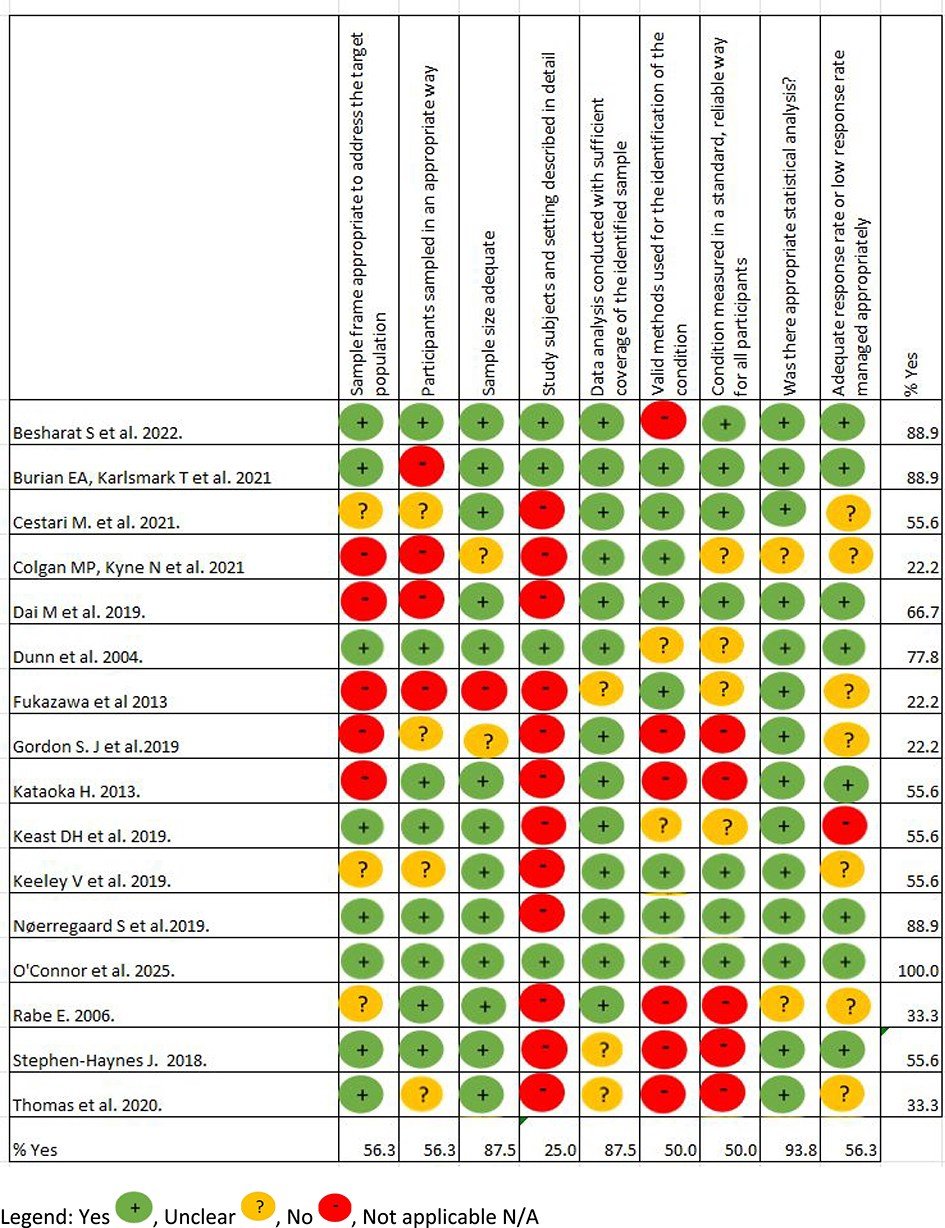

### Data synthesis and statistics

Prevalence data were summarised and presented as proportions (*n*, %) with 95% confidence intervals where reported, and were stratified by setting, including community population-based, non-population-based outpatient/specialist services and aged-care homes. Characteristics of older adults with chronic lower-limb oedema, including demographic, clinical and functional features, were summarised using reported frequencies, proportions and ranges. Summary tables were developed to enhance readability and facilitate comparison across studies. Within summary tables, study sample sizes (*n*) were used to calculate percentages, summarised as minimum, median and maximum values. Comorbidities, conditions co-occurring with oedema and mobility level were reported when ≥5 studies reported the outcome.

Risk factors were synthesised narratively, with adjusted odds ratios, regression coefficients or other effect estimates reported when multivariable analyses were undertaken. Heterogeneity was identified across studies, including differences in study populations, settings, sampling approaches, definitions of chronic oedema and outcome assessment methods, which limited comparability of prevalence estimates. As a result, meta-analyses were unable to be performed.

## Results

### Study selection

**Figure 1:**
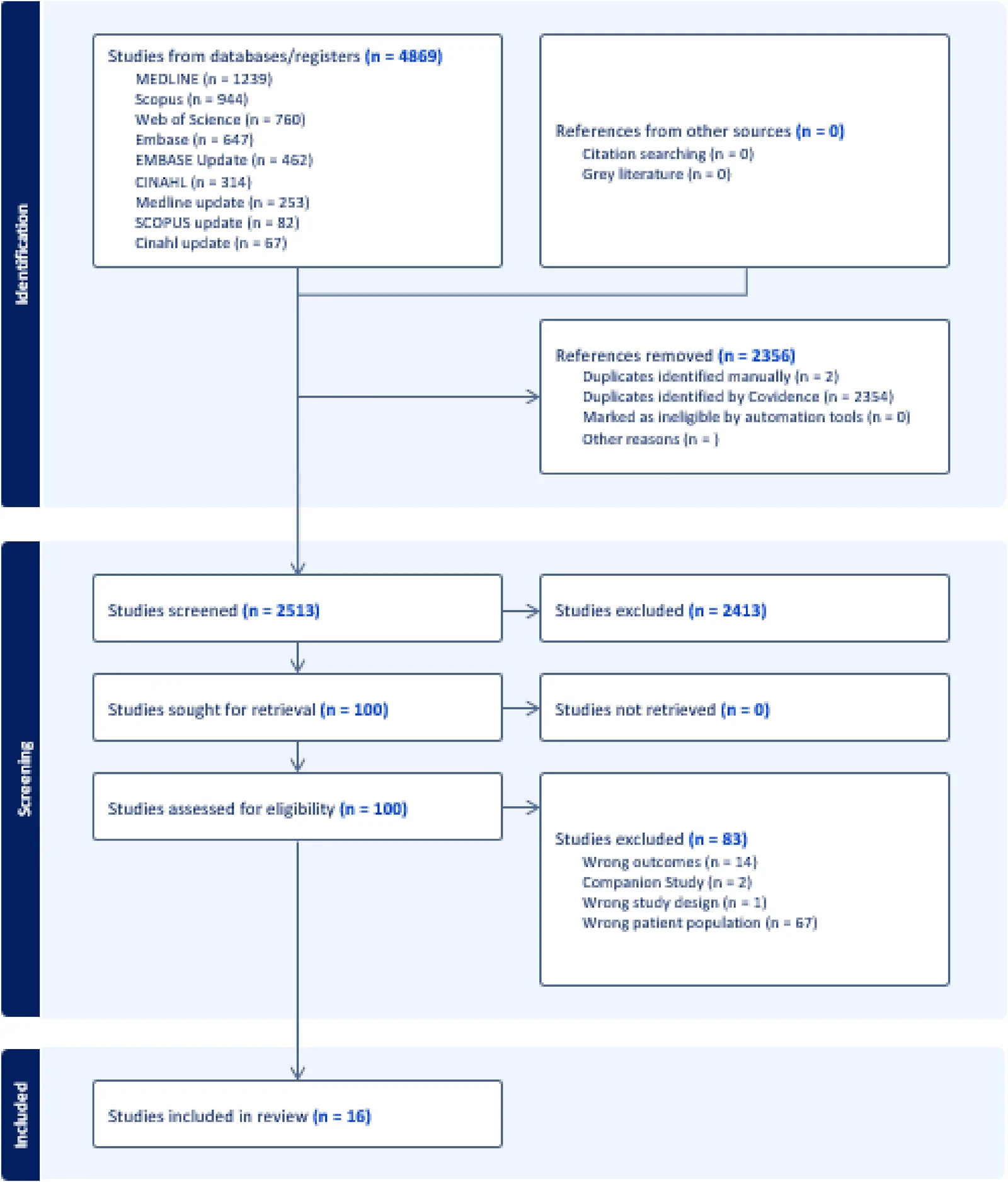
PRISMA flow chart.

The search yielded 4869 records leading to 100 studies for full text review. A further 84 studies were removed after full text review with 67 excluded due to wrong patient population, 14 due to wrong outcomes, two were companion studies and one study was excluded due to wrong study design (Figure 1).

### Quality assessment

Methodological quality for the included studies is presented in Table 1. Five studies met the threshold for low risk (≥70% Yes) [12, 13, 30–32], six for moderate risk (50–69% Yes) [21, 33–37] and five studies for high risk (<50% Yes) [17, 23, 38–40].

**Table 2:** Study outcome measurement.

Characteristic	Studies *n* (%)
Leg oedema terminology	
Chronic oedema defined as minimum 3 months duration	7 (43.8)
Chronic oedema with no defined duration	4 (25.0)
Lymphoedema/chronic oedema	1 (6.2)
Leg oedema	4 (25.0)
Assessment tool	
Pitting test	12 (75.0)
Questionnaire	2 (12.5)
Interview	1 (6.3)
Identification method not stated	2 (12.5)

Three JBI criteria were rated low risk (≥70% Yes): sample size adequacy (87.5%), data analysis coverage (87.5%) and appropriate statistical analysis (93.8%). Moderate risk (50–69% Yes) was identified for sample frame appropriateness, sampling method, response rate (or management of low response), validity of condition identification and reliability of measurement (50.0%–56.3%). The description of study subjects and setting was 25.0% rated high risk (<50% Yes).

### Study characteristics, recruitment and outcome measurement

Characteristics of the 16 included studies are summarised in Table 2, with additional details provided in Appendix S2 (See Supplementary Data section). Studies were published from 2004 to 2025. Three (19%) studies were based in the UK [13, 23, 37] with Australia (13%) [17, 32], Japan (13%) [34, 40] and the USA (13%) [12, 30] each having two studies. One study was published in Japanese.

Three studies employed community population-based sampling designs [12, 30, 39], with 11 studies using outpatient or specialist services, enrolling participants from a wide range of specialist services including oncology, vascular, wound, geriatric, cardiovascular and lymphoedema. Two studies included aged-care home settings, where participant identification occurred through staff-completed questionnaires or through on-site resident assessments with discretionary exclusion of residents who were unwell, asleep or unable to participate.

The pitting test was the commonly used outcome to measure oedema (*n* = 12, 75%). Reliability of the outcome measure was established in seven (43.8%) studies. Eight (50%) studies staged oedema severity, including five (31.3%) that used the International Society of Lymphology classification [13, 21, 31, 34, 36]. Nine (56.3%) studies mapped the distribution of oedema across multiple anatomical pitting sites along the leg [13, 17, 21, 31, 33, 34, 36, 38, 40].

Eight papers were identified during full-text screening associated with the Lymphoedema Impact Prevalence INTernational (LIMPRINT) study, a large international epidemiological study, designed to measure the size, impact and burden of chronic oedema across all age ranges and diverse health systems worldwide. Study authors contributed eligible data for nine countries for this systematic review with a smaller sample size than reported in the published LIMPRINT. The nine countries were Australia [17], Canada [21], Denmark [31], France [36], Ireland [38], Italy [33], Japan [34], UK (informed by Burian *et al*. [13] and Turkey [36]). Additionally, lower-limb-specific chronic oedema data were provided for one aged-care home study [23].

### Lower-limb oedema prevalence, participant characteristics and risk factors

The prevalence of lower-limb oedema was reported in 13 studies, consisting of three community population-based, eight non-population-based outpatient or specialist services and two aged-care home studies (Table 3 and Appendix S3; see Supplementary Data section). Community population-based prevalence were 22.7% [39], 23.7% [12] and 26.4% [30]. Non-population community outpatient and specialist settings prevalence estimates of lower-limb oedema ranged from 11.9% [35, 38] to 70.1% [13]. In aged-care home settings, lower-limb oedema prevalence ranged from 10.0% [37] to 27.5% [23].

**Table 3:** Summary of participant characteristics among older adults with lower-limb oedema from studies reporting prevalence data.

Variable	Studies *n* (%)	Participants *n*	Prevalence with condition (%)			Prevalence with no condition (%)		
			Min	Median	Max	Min	Median	Max
Total studies	13 (100)	142–13 098						
Prevalence studies								
Community population	3 (23.1)	784–13 098	22.7	23.7	26.4			
Community outpatient/specialist services	8 (61.5)	109–4606	11.9	38.4	70.1			
Aged-care homes	2 (15.4)	750–946	10.0	18.7	27.5			
Gender								
Female	9 (69.2)	61–3078	11.5	37.5	73.6	7.8	35.4	63.0
Male	9 (69.2)	48–1451	7.8	35.4	61.0	11.5	37.5	74.2
Comorbidity								
Diabetes	7 (53.8)	2–1190	14.5	60.0	100	10.3	37.3	72.4
Heart disease	7 (53.8)	3–1073	7.3	51.2	100	9.4	37.1	80.0
Neurological disease	5 (38.5)	28–466	4.2	46.4	65.0	23.6	41.3	70.6
Peripheral arterial disease	5 (38.5)	0–146	0	42.5	87.6	4.9	42.1	71.0
Normal weight	5 (38.5)	10–1747	32.3	55.1	64.0	6.1	24.7	79.2
Obesity/morbid obesity	5 (38.5)	5–2674	61.6	77.4	100	7.7	37.8	52.5
Co-occurring clinical condition								
Cellulitis	5 (38.5)	13–1917	72.5	92.3	100	25.7	37.3	68.4
Wounds	6 (46.2)	11–1468	0.6	66.9	100	23.7	37.7	89.1
Mobility level								
Walks unaided	6 (46.2)	40–1652	24.1	55.9	97.5	9.7	42.5	66.1
Walks aided	7 (53.8)	22–2297	30.7	62.0	70.5	17.5	26.1	72.4
Chairbound	6 (46.2)	9–513	20.4	56.5	81.8	20.7	38.9	70.2

Note: Table 3 summarises data from Appendix S3. Studies *n* (%) indicates the number of contributing studies and Participants *n* indicates the range of participant numbers. Percentages are summarised as the minimum, median and maximum prevalence among participants with lower-limb oedema.

Characteristics of older adults with lower-limb oedema were reported in 13 studies, 11 in community settings. The age of older adults ranged from 60 to over 90 years. The proportion of women with lower-limb oedema ranged from 15% to 89%, compared with 11% to 85% for men (Appendix S3 see Supplementary Data section). Lower-limb oedema was more common in those with diabetes, heart disease, obesity, cellulitis and wounds (Table 3 and Appendix S3). Lower-limb oedema was observed across all reported mobility categories (Table 3 and Appendix S3).

Risk factors of lower-limb oedema were examined in three studies (Appendix S4 see Supplementary Data section). Two studies performed logistic regression [32, 35] identifying statistically significant odds ratios for lower-limb oedema of varicose veins in the leg [35], heart disease and use of a mobility aid [32]. Multivariable linear regression was conducted in one study [40] reporting statistically significant coefficients for four variables of diabetes, lower-limb varicose veins, daytime activity level and low albumin.

## Discussion

Chronic lower-limb oedema prevalence among older adults is ~24% in community population-based studies and from 11.9% to 70.1% in non-population-based studies (outpatient/specialist services). Evidence from aged-care homes remains sparse and operationally constrained, suggesting that prevalence in this high-risk population may be underestimated. Reported prevalence varied according to assessment method (self-report vs clinical examination) and sampling frame (population vs outpatient vs service audit). Chronic lower-limb oedema occurs in the context of multimorbidity, particularly diabetes, heart failure and obesity, with a high proportion of individuals with chronic lower-limb oedema classified as obese or morbidly obese (median 77.4% across studies), and oedema is frequently reported alongside cellulitis, wounds and functional limitations. Evidence to support independent risk factors is limited. This systematic review is, to our knowledge, the first to report prevalence estimates, characteristics and potential risk factors for chronic lower-limb oedema among older adults (≥60 years) in community and aged-care home settings. Population-based prevalence estimates carried the highest confidence due to low risk of bias, while findings from outpatient/specialist services and aged-care populations were interpreted more cautiously due to potential selection and measurement bias.

Population-based studies suggest approximately one-quarter of older adults living in the community have chronic lower-limb oedema [12, 30, 39]. While only three population-based studies were identified for inclusion, the relative consistency of the reported prevalence is of note. Of these, two studies used the pitting test to determine the presence of lower-limb oedema [30, 39] and the remaining assessed lower-limb oedema via self-report [12]. Prevalence may be underestimated in this latter study, though it comprised a large, nationally representative cohort [12], which likely limits the impact of random misclassification on overall estimates. Similar prevalence rates have been previously reported when the pitting test was used, including a US community study reporting 26% [30] and a German population-based study reporting 23% [39].

A higher prevalence was found in specific populations, ranging from outpatient clinics to community aged-care providers. This may be due to these subgroups of older adults exhibiting high levels of clinical impairment, which likely elevates their risk of lower-limb oedema. Prevalence in aged-care homes remains unclear due to recruitment challenges and selection bias. With the global population aged 60 years and over expected to increase from ~1.1 to 1.4 billion by 2030 [18], chronic lower-limb oedema in older adults represents a substantial potential worldwide health and economic burden.

Characteristics co-occurring with lower-limb oedema were grouped into three domains: comorbidity-related factors, co-occurring clinical conditions and functional characteristics. Among community-dwelling older adults, diabetes, cardiovascular disease and obesity were comorbidities consistently co-occurring with lower-limb oedema, reflecting their shared effects on vascular and lymphatic fluid regulation [41–44]. This relationship is supported by a population-based study reporting increased odds of lower-limb oedema in individuals with diabetes (OR 1.22) and obesity (OR 2.33) [12]. A high prevalence of obesity was reported among individuals with lower-limb oedema. Although the available evidence does not establish an independent association, these findings suggest that screening for lower-limb oedema in older adults with obesity may merit further investigation.

Clinically, management of chronic lower-limb oedema in older adults is important due to its association with co-occurring conditions that increase morbidity, functional decline and healthcare use. Older adults with lower-limb oedema consistently experience higher rates of cellulitis than those without oedema, reflecting the bidirectional relationship between lymphatic failure and recurrent infection [13]. Cellulitis reduces quality of life and contributes to increased hospitalisation [14] and persistent mobility limitations [15]. Wounds, including venous leg ulcers, are also common in this population and further contribute to morbidity through pain, limb heaviness and reduced function [15]. Venous leg ulcer incidence ranges from 4% to 5% in the over-65 population, increasing approximately four-fold among those aged over 80 [45]. These complications impose a substantial economic burden, with chronic wounds accounting for an estimated 2%–4% of national health budgets in high-income countries [46]. Effective management of chronic lower-limb oedema reduces cellulitis recurrence and is less costly than treating recurrent infections [11].

An interesting finding of this systematic review was that lower-limb oedema appeared to be equally common among older adults who ambulated unaided, used a mobility aid or were chairbound. This pattern was consistent across community-based prevalence studies and specialist chronic-oedema outpatient cohorts. The reason for this is unclear. In chairbound individuals, prolonged dependency of the lower limbs and reduced calf muscle pump activation may impair venous and lymphatic return [47]. Among older adults using mobility aids, altered gait patterns, slower walking speed and lower levels of physical activity may contribute to impaired fluid transport [48]. Conversely, older adults may develop oedema primarily due to underlying comorbidities such as venous disease, heart disease, obesity or lymphatic dysfunction rather than mobility limitation itself. These findings suggest that broad mobility categories may not adequately capture differences in gait efficiency, calf muscle pump function, physical activity levels or sedentary behaviour, and future research should examine these factors directly.

Evidence of the prevalence and characteristics of lower-limb oedema in aged-care homes was limited and appears constrained by operational factors. Two studies [23, 37] used staff-mediated identification and discretionary inclusion of residents, processes that could contribute to under-ascertainment. Mild oedema may be missed, and unwell or unavailable residents may be excluded. Although this was not directly evaluated in the included studies, these methodological features suggest that facility-level prevalence may be underestimated. Notably, neither study aimed to measure prevalence, characteristics or risk factors. Further research is needed to establish the true prevalence of lower-limb oedema in aged-care homes.

While many of the reported characteristics are clinically recognised, the evidence supporting independent risk factors remains limited. Only three studies undertook multivariable analyses, with each examining different predictors and outcomes, limiting comparability and restricting conclusions regarding independent determinants of lower-limb oedema. Overall, heterogeneity in analytical approaches underscores the need for well-designed studies using standardised measures to enable more robust identification of independent determinants of lower-limb oedema.

### Limitations and future research

Several limitations should be considered when interpreting these findings. Study-level limitations included a lack of prospective and longitudinal studies. Substantial heterogeneity was observed across included studies in sampling frames, settings and population characteristics. Definitions of chronic lower-limb oedema varied, including in terminology, duration criteria and assessment methods. These factors, together with potential selection bias in service-based studies, likely contributed to variation in prevalence estimates. Additionally, complete reporting of participant characteristics, comorbidities and potential risk factors was often lacking. Finally, evidence from aged-care homes was limited with reliance on staff-mediated identification and selective resident participation, potentially leading to under-ascertainment of oedema and reduced confidence in estimates for this high-risk population. Collectively, these limitations reduce confidence in prevalence estimates for community service-based and aged-care home studies.

A meta-analysis was not undertaken in this review due to methodological and clinical heterogeneity; findings were therefore narratively reported. Outpatient and specialist service populations were combined into a non-population-based category, as some studies reported pooled data from multiple settings. A three-way stratification by community, outpatient and specialist setting was not feasible without either arbitrarily reassigning mixed-setting studies to a single category or fragmenting the data into subgroups too small for meaningful comparison. Randomised controlled trials were excluded, although this may have limited access to additional baseline participant characteristic data.

Future research should prioritise methodologically robust, prospective longitudinal population-based epidemiological studies of older adults across community and aged-care settings, using standardised and validated assessment methods for oedema. Comprehensive and standardised reporting of comorbidity, functional status, frailty and risk factors is needed to clarify the relative contributions of cardiovascular, metabolic, and mobility-related factors. Longitudinal research is needed to examine temporal associations between lower-limb oedema and multimorbidity, immobility, obesity and lymphatic dysfunction, and the risk of subsequent complications, including cellulitis and chronic wounds. Addressing these evidence gaps would improve the certainty and applicability of findings and inform earlier recognition and more targeted risk reduction strategies for chronic lower-limb oedema in ageing populations.

## Conclusion

Chronic lower-limb oedema is frequently reported among older adults and represents a potentially significant under-recognised health issue across community and aged-care settings. The condition commonly co-occurs with multimorbidity, particularly diabetes, heart failure and obesity, and is linked to complications including cellulitis and wounds. Despite this potential clinical and economic burden, the evidence base, particularly within aged-care homes, remains limited and heterogeneous.

Greater clinical awareness of chronic lower-limb oedema and its associated complications may support earlier identification and management in older adults, particularly those with multimorbidity. Further research using standardised assessments and more comprehensive reporting is needed to better characterise chronic lower-limb burden and inform targeted case-finding. Embedding lower-limb oedema screening into existing chronic disease review consultations, rather than waiting for oedema to present symptomatically, may represent a pragmatic, low-cost opportunity for earlier detection in both community and aged-care settings.

## Supplementary Material

Supplementary_material_afag277
