# Supplementary material for: Prevalence, characteristics and risk factors for chronic lower-limb oedema in the older population living in the community or in an aged-care home: a systematic review
Source: Age Ageing. 2026 Sep 22;55(9):afag277. doi: 10.1093/ageing/afag277 (PMC13596240; doi:10.1093/ageing/afag277)
Supplement: Supplementary file 1 [file supplementary_material_afag277.zip › aa-26-1432-File002_afag277.docx]

**Prevalence, characteristics and risk factors for** **chronic lower limb oedema in the older population living in the community or in an aged-care home: A systematic review.**

**Supplementary data**

Appendix 1 – Medline search strings

Appendix 2 - Study characteristics, age, gender and chronic lower limb oedema prevalence, cause, duration, severity and measurement method

Appendix 3 - Characteristics of chronic lower limb oedema

Appendix 4 - Results from studies that performed logistic regression

Appendix 1 Medline search strings

| Search | Search Term |
| --- | --- |
| 1 | TI (“older adult*” OR elderly OR aged OR geriatric* OR “old age” OR retire*) OR  AB (“older adult*” OR elderly OR aged OR geriatric* OR “old age” OR retire*) |
| 2 | (MH "Aged") OR (MH "Frail Elderly") |
| 3 | TI (chronic OR persistent) OR AB (chronic OR persistent) |
| 4 | TI (oedema OR edema OR Lymphedema OR Lymphoedema) OR AB (oedema OR edema OR Lymphedema OR Lymphoedema) |
| 5 | MH "Edema") OR (MH "Lymphedema") |
| 6 | S4 OR S5 |
| 7 | TI (“lower limb*” OR leg* OR peripheral*) OR AB (“lower limb*” OR leg* OR peripheral*) |
| 8 | (MH "Lower Extremity") |
| 9 | S7 OR S8 |
| 10 | S1 OR S2 |
| 11 | S3 AND S6 AND S9 AND S10 |

Appendix 2 Study characteristics, age, gender and chronic lower limb oedema prevalence, cause, duration, severity and measurement method

| **Author/Year/**  **Country/Design** | **No oedema (n, %)**  **Age (Years) (Mean +/- SD)/ Median (IQR/Range), Gender (n, %)** | **Prevalence Oedema (n, %, 95%CI)**  **Age (Years) (Mean +/-SD)/ Median (IQR/Range), Gender (n, %) Unilateral/Bilateral (n, %** | **Cause/Duration/Severity n (%)** | **Measurement method (reliability*)/ Staging** |
| --- | --- | --- | --- | --- |
| *Community - population* | | | | |
| Besharat S et al. 2022, United States, prospective longitudinal | n = 9995 (76.3)  Age:  60 to 69: n = 4760  70 to 79: n = 3187  80 to 89: n = 1786  90+: n = 262  P<0.0001  Gender- not stated for 60+ years | Prevalence: n = 3103 (23.7)  Age:  60 to 69: n = 1136  70 to 79: n = 1025  80 to 89: n- = 83  90+: n = 159  P<0.0001  Gender- not stated for 60+ years  Unilateral/bilateral: not stated | Cause: not stated  Duration: not stated  Severity: not stated | Measurement: Interview (telephone or in person)  Staging: not stated |
| Dunn et al. 2004, United States, prospective observational | n = 577 (73.6)  Age (entire cohort): 74.5 +/- 6.03  Gender (entire cohort): male n = 339 (43%), female n=445 (57%) | Prevalence: n= 207 (26.4)  Severity (significant) = 19  Age – not stated  Gender- male = 22.6%, female n= 28.8%, p = 0.139 | Cause: not stated  Duration: not stated  Severity (significant): 9.0% | Measurement: Pitting test  Staged: None, visual swelling, pitting, or marked; pitting or marked oedema was considered clinically significant. |
| Rabe E. 2006, Germany, prospective cohort | n = 686 (77.3)  Age:  60-69 =442 (49.8)  70-79 =244 (27.5)  Gender: not stated for age 60+ | Prevalence: n = 202 (22.7)  Age:  60-69=118 (13.3)  70-79=84 (9.5)  Gender: not stated for age 60+ | Cause: not stated  Duration: not stated  Severity: not stated | Measurement: Pitting oedema CEAP classification C3 indicates oedema  Staging: Not stated |
| Community – outpatient/specialist services | | | | |
| Burian EA, Karlsmark T et al. 2021, United Kingdom prospective cross sectional ^ | n = 1379 (29.9)  Age: 79.6 (9.4)  Female = 81.2 +/- 9.1  Male = 77.3 +/- 9.3  Gender: female = 813 (17.7), male = 566 (12.3) | Prevalence: n = 3227 (70.1)  Age: 74.5 +/- 9.0  Female = 74.8 +/- 9.0  Male = 73.7 +/- 8.7  Gender: female = 2265 (49.2), male = 885 (19.2)  Unilateral: 440 (13.6)/ Bilateral: 2787 (86.4) | Cause: venous = 1102 (34.1), primary = 315 (9.8), cancer = 422 (13.1)  Duration:  <6 months = 90 (2.8)  6-12 months= 132 (4.1)  1-2 years = 285 (8.8)  2-5 years = 699 (21.7)  5-10 years = 858 (26.6)  >10 years = 1160 (35.9)  Severity:  ISL Stage I = 98  ISL Stage II = 202  ISL Stage III = 47 | Measurement: Questionnaire and clinical examination  Pitting test*  Staging: ISL Staging+ |
| Cestari M. et al. 2021, Italy, prospective cohort ^  All participants with oedema | Not applicable | Prevalence: not applicable (oedema service)  N=595  Age: 73.2 +/-7.5  Female 73.1 +/- 7.4  Male 76.4 +/- 7.9  Gender: female n=449 (75.5), male n=145 (24.4)  Unilateral: 230 (38.7)/ Bilateral: 365 (61.3) | Cause: venous = 232 (39.0), primary = 133 (22.4), cancer = 138 (23.2)  Duration:  <6 months = 42 (7.1)  6-12 months=57 (9.6)  1-2 years = 73 (12.3)  2-5 years = 151 (25.4)  5-10 years = 109 (18.3)  >10 years = 163 (27.4)  Severity: not stated | Measurement: Pitting test*  Staging: Not stated |
| Colgan MP, Kyne N et al. 2021, Ireland, prospective cross sectional^ | n = 96 (88)  Age: 73.2 (8.9)  Age: female = 75.3 (9.0), male = 70.4 (8.0)  Gender: female = 54 (49.5), male = 42 (38.5) | Prevalence: n = 13 (12.0)  Age: 75.7 (9.4)  Age: female = 74.7 (9.0), male = 76.8 (8.2)  Gender: female = 7 (6.4), male = 6 (5.5)  Unilateral: 4 (38.8) / Bilateral: 9 (69.2) | Cause: venous = 0, primary = 5 (38.5), cancer = 3 (23.1)  Duration:  <6 months = 1 (7.7)  6-12 months= 0  1-2 years = 0  2-5 years = 2 (15.4)  5-10 years = 5 (38.5)  >10 years = 5 (38.5)  Severity:  ISL Stage I = 1  ISL Stage II = 2  ISL Stage III = 5 | Measurement: Pitting test*  Staging: ISL Staging+ |
| Dai M et al. 2019, Japan, prospective cross sectional^ | n = 253  Age: 86.4 +/- 7.4  Female = 87.4 +/- 7.0  Male = 84.0 +/- 7.6  Gender: female = 176 (55.2), male = 76 (23.8) | Prevalence: n = 66 (20.7)  Age: 77.9 +/- 9.2  Female = 78.0 +/- 9.4  Male = 77.2 +/- 8.3  Gender: female = 56 (17.6), male = 10 (3.1)  Unilateral: 36 (54.5) / Bilateral: 30 (45.5) | Cause: venous = 3 (4.5), primary = 0, cancer = 35 (53.0)  Duration:  <6 months = 9 (13.6)  6-12 months= 6 (9.1)  1-2 years = 5 (7.6)  2-5 years = 11 (16.7)  5-10 years = 15 (22.7)  >10 years = 20 (30.3)  Severity:  ISL Stage I = 1  ISL Stage II = 36  ISL Stage III = 2 | Measurement: Pitting test*  Staging: Not stated |
| Fukazawa R et al. 2013, Japan, prospective cohort | n = 87 (61.3)  Age: 82.6 +/- 6.7 p<0.05  Gender: male n=42 (29.6)  , female n=45 (31.7), not significant | Prevalence: n = 55 (38.7)  Oedema =36  Age: 84.5 +/-6.5;  Very mild n = 19  Age: 86.6 +/- 4.6, p<0.05  Gender:  Male oedema n=20 (14.1), mild oedema n=8 (5.7)  Female oedema n=16 (11.3), mild oedema n=11 (7.7), not significant | Cause: not stated  Duration: not stated  Severity: very mild 19% (34.5) | Measurement: Comorbidities and lifestyle - interview  Oedema- observation and pitting test  Staging: no oedema, mild oedema, oedema |
| Gordon S. J et al.2019, Australia, prospective cross sectional ^ | n = 90 (51.7)  Age: 79.1 +/-9.1  Female = 79.4 +/-9.3  Male = 78.6 +/- 8.8  Gender: female = 56 (32.2), male = 34 (19.5) | Prevalence: n = 84 (48.3)  Age: 79.5 (9.9)  Female = 81.8 +/-9.0  Male = 75.3 +/- 10.2  Gender: female = 54 (31.0), male = 30 (17.2)  Unilateral: 12 (14.3) / Bilateral: 72 (85.7) | Cause: venous = 56 (66.7), primary = 9 (10.7), cancer = 4 (4.8)  Duration:  <6 months = 16 (19.0)  6-12 months= 9 (10.7)  1-2 years = 12 (14.3)  2-5 years = 14 (16.7)  5-10 years = 11 (13.1)  >10 years = 22 (26.2)  Severity:  ISL Stage I = 36  ISL Stage II = 30  ISL Stage III = 15 | LIMPRINT assessment tools> |
| Kataoka H 2013, Japan, prospective cohort | n = 241 (88.0)  Age: 68.5 +/-11.5 p=0.006  Gender: male n=71 (25.9), female n=170 (62.0), p=0.18 | Prevalence: n = 33 (12.0, 8.2-15.9)  Age: 74.4 +/- 10.7 p=0.006  Gender: male n=6 (2.2), female n=27 (9.9), p=0.18  Unilateral: 0 / Bilateral: 33 (100.0) | Cause: not stated  Duration: not stated  Severity: not stated | Measurement: Not stated  Staging: Severity graded 1- 4 based on location.  (1) none, (2) up to ankle level height (mild oedema), (3) up to knee height (moderate oedema), (4) higher than knee level (severe oedema). |
| Keast DH et al. 2019, Canada, prospective cohort^  (All participants with oedema) | Not applicable | Prevalence: not applicable (oedema service)  N = 54  Age:70.6 +/- 8.3  Female = 71.6 +/- 8.8  Male = 69.9 +/- 8.1  Gender: female n = 23 (42.6), male n= 31 (57.4)  Unilateral: 6 (11.1) /Bilateral: 48 (88.9) | Cause: venous = 40 (74.1) primary = 3 (5.6), cancer = 6 (11.1)  Duration:  <6 months = 1 (1.9)  6-12 months= 2 (3.7)  1-2 years = 0  2-5 years = 7 (13.0)  5-10 years = 15 (27.8)  >10 years = 29 (53.7)  Severity:  ISL Stage I = 8  ISL Stage II = 35  ISL Stage III = 11 | Measurement: Pitting test*  Staging: ISL Staging+ |
| Keeley V et al. 2019, France & Turkey only, retrospective cross sectional^  Turkey: All participants with oedema | *France* | | | |
|  | n = 305 (57.5)  Age: 77.0 +/- 10.0  Female = 79.6 +/-10.8  Male = 75.3 +/- 8.8  Gender: female = 158 (29.8), male = 147 (27.7) | Prevalence: n = 225 (42.5)  Age: 75.3 +/- 9.9  Female = 75.2 +/-10.2  Male = 75.5 +/- 5  Gender: female = 131 (22.8), male = 94 (17.9)  Unilateral: 67 (29.8) / Bilateral: 158 (70.2) | Cause: venous = 84 (37.3), primary = 34 (15.1), cancer = 38 (16.9)  Duration:  <6 months = 37 (16.4)  6-12 months=15 (6.7)  1-2 years = 18 (8.0)  2-5 years = 43 (19.1)  5-10 years = 38 (16.9)  >10 years = 63 (28.0)  Severity: not stated | Measurement: Pitting test*  Staging: Not stated |
|  | *Turkey* | | | |
|  | Not applicable | Prevalence: not applicable (oedema service)  N=75  Age: 68.3 +/- 6.8  Female = 67.9 +/- 6.4  Male = 71.8 +/- 8.9  Gender: female = 67 (89.3), male = 8 (10.7)  Unilateral: 30 (40.0)/ Bilateral: 45 (60) | Cause: venous = 24 (32.0), primary = 8 (10.7), cancer = 31 (41.3)  Duration:  <6 months = 5 (6.7)  6-12 months = 8 (10.7)  1-2 years = 12 (16.0)  2-5 years = 25 (33.3)  5-10 years = 7 (9.3)  >10 years = 18 (24.0)  Severity:  ISL Stage I = 11  ISL Stage II = 43  ISL Stage III = 18 | Measurement: Pitting test*  Staging: ISL Staging+ |
| Nøerregaard S et al.2019, Denmark, prospective cohort^  (All participants with oedema) | Not applicable | Prevalence: not applicable (oedema service)  N=636  Age: 76.4 +/- 9.4  Female = 78.1 +/- 9.4  Male = 74.6 +/- 9.0  Gender: female = 337 (53.0), male = 299 (47.0)  Unilateral: 157 (24.7) / Bilateral: 479 (75.3) | Cause: venous = 417 (65.6), primary = 22 (3.5), cancer = 18 (2.8)  Duration:  <6 months = 88 (13.8)  6-12 months = 90 (14.2)  1-2 years = 114 (17.9)  2-5 years = 136 (21.4)  5-10 years = 75 (11.8)  >10 years = 133 (20.9)  Severity:  ISL Stage I = 31  ISL Stage II = 24  ISL Stage III = 17 | Measurement: Pitting test*  Staging: ISL Staging+ |
| O'Connor M et al. 2025, Australia, retrospective cohort | n = 284 (61.9)  Age: 80.7 +/- 7.2, p=0.154  Gender: male 93 (20.3), female 191 (41.6), p=0.419 | Prevalence: n = 175 (38.1)  (bilateral = 150, 85.7%)  Age: 81.0 +/- 7.7, p=0.154  Gender: male 51 (11.1), female 124 (27.) | Cause: Not stated  Duration: not stated  Severity: not stated | Measurement: Pitting test  Staging: Not stated |
| *Aged-care homes* | | | |  |
| Stephen-Haynes J. 2018, United Kingdom, prospective audit | n = 675 (90.0)  Age: not stated (author confirmed ≥60 years)  Gender: not stated | Prevalence: n = 75 (10.0)  Age: not stated (author confirmed ≥60 years)  Gender: not stated | Cause: not stated  Duration: not stated  Severity: not stated | Measurement: questionnaire  Staging: Not stated |
| Thomas M. et al. 2020, United Kingdom, prospective cross sectional # | n = 686 (72.5)  Age:  60-69: n = 26 (2.7)  70-79: n = 124 (13.1)  80-89: n = 318 (33.6)  90+: n = 218 (23.0)  Gender: male n = 157 (16.6), female n=488 (51.6) | Prevalence: n = 260 (27.5)  Age:  60-69: n=12  70-79: n=43  80-89: n=111  90+: n=94  Gender: male n=60, female n=198 | Cause: not stated  Duration: not stated  Severity:  Mild = 63.8% (n=166)  Moderate = 23.8% (n=62)  severe/significant = 6.15%  (n=16)  Complex = 6.15% (n=16) | Measurement: Not stated  Staging: LNW Outcome Severity Scale* |

^Data ≥60 years provided from LIMPRINT authors

\# Data ≥60 years provided by author

+ International Society of Lymphology (ISL)

*Lymphoedema Network Wales (LNW) Outcome Severity Scale

> Lymphoedema IMpact and PRevalence— INTernational (LIMPRINT) assessment tool

Appendix 3 Characteristics of chronic lower limb oedema

| **Author/Year** | **Neurological/**  **Dementia**  **n (%)** | **Heart disease/ hypertension/ diabetes/ weight**  **n (%) /Mean +/- SD** | **Venous disease/ peripheral arterial disease (PAD)/wound/pressure injury/cellulitis n (%)** | **Mobility/activity level/ joint disorders/ pain**  **n (%)** | **Medication/**  **Polypharmacy**  **n (%) /Mean (SD)** |
| --- | --- | --- | --- | --- | --- |
| *Community - population* | | | | | |
| Besharat S et al. 2022 | Not stated | Heart disease: Not stated  Hypertension/diabetes/  Weight: Not available for 60 years and older | Not stated | Mobility: Not stated  Activity and Pain: Not available for 60 years and older | Not available for 60 years and older |
| Dunn et al. 2004 | Not Stated | Not Stated | Not Stated | Not Stated | Not Stated |
| Rabe E 2006 | Not Stated | Not Stated | Not Stated | Not Stated | Not Stated |
| Community – outpatient/specialist services | | | | | |
| Burian E et al. 2021 | Neurological:  No oedema = 163  Oedema =303  Dementia: Not stated | Heart Failure:  No oedema = 402  Oedema = 671  Hypertension: Not stated  Diabetes:  No oedema = 437  Oedema = 753  Weight:  Underweight  No oedema = 134  Oedema = 51  Normal  No oedema = 784  Oedema = 963  Obese  No oedema = 389  Oedema = 1389  Morbidly obese  No oedema = 72  Oedema = 824 | Venous disease:  No oedema= not stated  Oedema = 1102  PAD:  No oedema = 84  Oedema = 62  Wound:  No oedema = 1036  Oedema = 432  Leg ulcer:  No oedema = 607  Oedema = 244  Pressure ulcer:  No oedema = 122  Oedema = 37  Cellulitis:  No oedema = 528  Oedema = 1389 | Mobility:  Bedbound  No oedema = 100  Oedema = 43  Chairbound  No oedema =161  Oedema = 352  Walks with aid  No oedema = 742  Oedema = 1555  Walks unaided  No oedema =376  Oedema =1276  Activity level: Not stated  Joint disorders/ pain: Not stated | Not stated |
| Cestari M. et al. ^  (All participants with oedema) | Neurological: 14  Dementia: not stated | Heart failure: 74 (%)  Hypertension: not stated  Diabetes: n=71  Weight:  Underweight n= 10  Normal n= 415  Obese n= 164  Morbidly obese- 5 | Venous disease: 232  PAD:20  Leg ulcer: 91  Pressure ulcer: 0  Cellulitis: 160 | Mobility:  Bedbound =0  Chairbound =12  Walks with aid =99  Walks unaided =484  Activity level: Not stated  Joint disorders/pain: Not stated | Not Stated |
| Colgan MP, Kyne N et al. 2021 ^ | Neurological:  No oedema = Not stated  Oedema- 0  Dementia: Not stated | Heart Failure:  No oedema = Not stated  Oedema =3  Hypertension: Not stated  Diabetes:  No oedema = Not stated  Oedema =2  Weight:  Underweight  No oedema-=1  Oedema =0  Normal  No oedema =3  Oedema =7  Obese  No oedema =0  Oedema =5  Morbidly obese-  No oedema =0  Oedema =0 | Venous disease:  No oedema= not stated  Oedema = 0  PAD:  No oedema = Not stated  Oedema =0  Leg ulcer:  No oedema = Not stated  Oedema =5  Pressure ulcer:  No oedema = Not stated  Oedema =0  Cellulitis:  No oedema = Not stated  Oedema =6 | Mobility:  Bedbound  No oedema = Not stated  Oedema =0  Chairbound  No oedema = Not stated  Oedema =0  Walks with aid  No oedema = Not stated  Oedema =9  Walks unaided  No oedema = Not stated  Oedema =4  Activity level: Not stated  Joint disorders/ pain: Not stated | Not stated |
| Dai M et al. 2019 ^ | Neurological:  No oedema =46  Oedema =2  Dementia: Not stated | Heart disease:  No oedema = 51  Oedema = 4  Hypertension: Not stated  Diabetes:  No oedema =47  Oedema =8  Weight:  Underweight-  No oedema-=153  Oedema =9  Normal-  No oedema =88  Oedema =42  Obese  No oedema =8  Oedema =12  Morbidly obese  No oedema =1  Oedema =3 | Venous disease: 3  PAD:  No oedema =7  Oedema =0  Wound:  No oedema =160  Oedema =1  Leg ulcer:  No oedema =4  Oedema =0  Pressure ulcer:  No oedema =9  Oedema =1  Cellulitis:  No oedema = not stated  Oedema =23 | Mobility:  Bedbound-  No oedema =205  Oedema =3  Chairbound-  No oedema =39  Oedema-=10  Walks with aid-  No oedema =8  Oedema =14  Walks unaided-  No oedema =1  Oedema =39  Activity level: Not stated  Joint disorders/ pain: Not stated | Not stated |
| Fukazawa R et al. 2013 | Cerebrovascular disease:  No oedema =23 (26.4)  Oedema =10 (27.8)  Very mild =4 (21.1)  p=not significant  Parkinson:  No oedema =8 (9.2)  Oedema =2 (5.6)  Very mild =2 (10.5)  p=not significant  Alzheimer’s:  No oedema =23 (26.4)  Oedema =7 (19.4)  Very mild =4 (21.1)  p=not significant | Heart disease:  No oedema =14 (16.1)  Oedema =10 (27.8)  Very mild =2 (10.5)  p=not significant  Atrial fibrillation:  No oedema =6 (6.9)  Oedema =7 (19.4)  Very mild =2 (10.5)  p<0.05  Hypertension:  No oedema =41 (47.1)  Oedema =23 (63.9)  Very mild =8 (42.1)  p<0.01  Diabetes:  No oedema =8 (9.2)  Oedema =11 (30.6)  Very mild =1(5.3)  p<0.01  Weight (kg):  No oedema =52.0 +/- 8.9  Oedema =58.0 +/-12.5  Very mild =51.9 +/- 10.1  p<0.05  Low albuminemia (<3.5g/dL):  No oedema =1 (1.1)  Oedema =5 (13.9)  Very mild =0  p<0.01 | Varicose veins (Grade 1/Grade 2)  No oedema =24/0  Oedema =11/7  Very mild =7  p<0.05  PAD, wound, pressure injury, cellulitis: Not stated | Mobility:  Independent-  No oedema =63 (72.4)  Oedema =14 (38.9)  Very mild =6 (31.6)  p<0.01  Walk with aid  No oedema =19 (21.8)  Oedema =19 (52.8)  Very mild =12 (63.2)  p<0.01  Chairbound-  No oedema =5 (5.7)  Oedema =3 (8.3)  Very mild =1 (5.3)  p<0.01  Activity level:  Active  No oedema 54 =(62.1)  Oedema 8 =(22.2)  Very mild =4 (21.1)  P=0.068  Sedentary  No oedema =26 (29.9)  Oedema =26 (72.2)  Very mild =13 (68.4)  p=0.068  Bedridden  No oedema =7 (8.0)  Oedema =2 (5.6)  Very mild =2 (10.5)  p=0.068  Frequency of going out (< 4/week):  No oedema =26 (29.9),  p<0.01  Oedema =22 (61.1) p <0.05  Very mild =9 (47.4), p <0.05  Joint disorders:  No oedema =22 (25.3)  Oedema =15 (41.7)  Very mild =7 (36.8)  p = not significant | Number of medications:  No oedema =5.1 +/- 2.7  Oedema =6.3 +/- 2.4  Very mild =5.5 +/- 2.7  p=0.078  Medication:  ARB  No oedema =25 (28.7)  Oedema =19 (52.8)  Very mild =5(26.3)  p<0.05  CCB  No oedema =27 (31.0)  Oedema =17 (47.2)  Very mild =6 (31.6)  p=0.68  Diuretics (for hypertension)  No oedema =11 (12.6)  Oedema =10 (27.8)  Very mild =4 (21.1)  p=not significant  PGZ (for diabetes)  No oedema =2 (2.3)  Oedema =4 (11.1)  Very mild =0  p=0.06  NSAID  No oedema =12 (13.8)  Oedema =4 (11.1)  Very mild =4 (21.1)  P= not significant  Polypharmacy (>7)  No oedema =22 (25.3)  Oedema =14 (38.9)  Very mild =6 (31.6)  p=0.099 |
| Gordon S. J et al.2019 ^ | Neurological:  No oedema =14  Oedema =13  Dementia: Not stated | Heart disease:  No oedema = 20  Oedema = 21  Hypertension: Not stated  Diabetes:  No oedema =11  Oedema =21  Weight:  Underweight-  No oedema-=9  Oedema =9  Normal-  No oedema =53  Oedema =30  Obese-  No oedema =27  Oedema =33  Morbidly obese-  No oedema =1  Oedema =12 | Venous disease:  No oedema =not stated  Oedema =56  PAD:  No oedema =4  Oedema =26  Wound:  No oedema =16  Oedema =61  Leg ulcer:  No oedema =1  Oedema =48  Pressure ulcer:  No oedema =1  Oedema =2  Cellulitis:  No oedema = 0  Oedema =25 | Mobility:  Bedbound-  No oedema =1  Oedema =0  Chairbound-  No oedema =2  Oedema-=9  Walks with aid-  No oedema =49  Oedema =48  Walks unaided-  No oedema =38  Oedema =27  Activity level: Not stated  Joint disorders/ pain: Not stated | Not stated |
| Kataoka H 2013 | Neurological/dementia: Not Stated | Heart disease: not stated (all patients at risk)  Hypertension/diabetes: Not stated  Weight:  Obesity (BMI ≥30 kg/m2)  No oedema =22 (9)  Oedema =5 (15)  p=0.34 | Venous disease:  Varicose veins  No oedema =39(15)  Oedema =19 (55)  p<0.001  PAD, wound, pressure injury and cellulitis: Not stated | Not stated | Medication  ACEI/ARB:  No oedema =85 (35)  Oedema =13 (39)  p=0.67  β-Blocker:  No oedema =91 (38)  Oedema =13 (39)  p=0.85  CCB:  No oedema =148 (61)  Oedema =20 (61)  p=1.00  Diuretics:  No oedema =29 (12)  Oedema =8 (24)  p=0.10  Diabetes meds:  No oedema =19 (8)  Oedema =5(15)  p=0.18  Polypharmacy: Not stated |
| Keast DH et al. 2019 ^  (All participants with oedema) | Neurological: 4  Dementia: Not stated | Heart failure: 13  Hypertension: Not stated  Diabetes: 25  Weight:  Underweight = 0  Normal = 12  Obese = 20  Morbidly obese =22 | Venous disease: 40  PAD: 2  Wound: 22  Leg ulcer:21  Pressure ulcer: 0  Cellulitis: 41 | Mobility:  Bedbound = 0  Chairbound =6  Walks with aid =30  Walks unaided =18  Activity level: Not stated  Joint disorders / pain: Not stated | Not stated |
| Keeley V et al. 2019 ^ | France | | | | |
|  | Neurological:  No oedema- 11  Oedema- 18  Dementia: Not stated | Heart Failure:  No oedema =28  Oedema =59  Hypertension: Not stated  Diabetes:  No oedema =33  Oedema =47  Weight:  Underweight-  No oedema-=14  Oedema =6  Normal  No oedema =86  Oedema =153  Obese  No oedema =13  Oedema =42  Morbidly obese  No oedema =1  Oedema =6 | Venous disease: 84  PAD:  No oedema = 18  Oedema = 16  Wound:  No oedema-=44  Oedema-=53  Leg ulcer:  No oedema =6  Oedema =16  Pressure ulcer:  No oedema =19  Oedema =16  Cellulitis:  No oedema =1  Oedema =44 | Mobility:  Bedbound-  No oedema= 26  Oedema =29  Chairbound-  No oedema =11  Oedema =33  Walks with aid-  No oedema =26  Oedema =62  Walks unaided-  No oedema-=41  Oedema =97  Activity level: Not stated  Joint disorders/pain: Not stated | Not stated |
|  | Turkey: (All participants with oedema) | | | | |
|  | Neurological: 3  Dementia: Not stated | Heart Failure:12  Hypertension: Not stated  Diabetes: 24  Weight:  Underweight- 2  Normal- 33  Obese-30  Morbidly obese- 10 | Venous disease: 24  PAD: 15  Wound: 5  Leg ulcer: 2  Pressure ulcer: 0  Cellulitis: 21  Admitted = 14 | Mobility:  Bedbound- 4  Chairbound- 2  Walks with aid- 16  Walks unaided- 53  Activity level: Not stated  Joint disorders/pain: Not stated | Not stated |
| Nøerregaard S et al.2019 ^  (All participants with oedema) | Neurological: 75  Dementia: Not stated | Heart failure: 231  Hypertension: Not stated  Diabetes: 158  Weight:  Underweight = 43  Normal = 383  Obese = 166  Morbidly obese = 44 | Venous disease: 417  PAD: 77  Wound: 240  Leg ulcer: 181  Pressure ulcer: 37  Cellulitis: 478 | Mobility:  Bedbound = 24  Chairbound = 68  Walks with aid = 254  Walks unaided = 290  Activity level: Not stated  Joint disorders/pain: Not stated | Not stated |
| O'Connor M et al. 2025 | Neurological: Not stated  Dementia:  No oedema =61 (21.5)  Oedema =26 (14.9)  p=0.079 | Heart disease:  No oedema = 62 (21.8)  Oedema = 86 (49.1)  p<0.001  Hypertension:  No oedema =191 (67.3)  Oedema =127 (72.6)  p=0.23  Diabetes:  No oedema =91 (32.0)  Oedema =60 (34.3)  p=0.619  Weight: Not stated | Venous disease/PAD: Not stated  Wound:  No oedema = 1 (0.4)  Oedema = 18 (10.3)  p<0.001  Pressure injury: Not stated  Cellulitis:  No oedema = 1 (0.4)  Oedema = 12 (6.9)  p<0.001 | Mobility:  Independent- Not stated  Walk with aid-  No oedema =169 (59.5)  Oedema =139 (79.4)  p<0.001  Activity level: Not stated  Joint disorders/pain: Not stated | Not stated |
| *Aged-Care Homes* | | | | | |
| Stephen-Haynes J. 2018 | Not stated | Not stated | Not stated | Not stated | Not stated |
| Thomas M. et al. 2020 # | Not stated | Not stated | Venous disease and PAD: Not stated  Wound:  No oedema =0  Oedema =11 (4.2)  Pressure injury: Not stated  Cellulitis:  No oedema =8 (1.1)  Oedema =25 (9.6) | Mobility:  Independent-  No oedema =93 (13.3)  Oedema =34 (13.1)  With aid  No oedema =189 (27.0)  Oedema =110 (42.3)  With assistance  No oedema =242 (34.6)  Oedema =81 (31.2)  Chairbound  No oedema =57 (8.1)  Oedema =28 (10.6)  Bedbound  No oedema =106 (15.1)  Oedema =7 (2.7)  Activity level: Not stated  Falls:  No oedema =13 (1.9)  Oedema =28 (10.8)  Joint disorders/pain: Not stated | Not stated |

Appendix 4 Results from studies that performed logistic regression

| **Author/Year** | **Age/Gender/Clinical Characteristics** | **Co-morbidities** | **Medication** | **Mobility/Exercise** |
| --- | --- | --- | --- | --- |
| Fukazawa R et al. 2013#  *(R = 0.671, adjusted R²＝ 0.365, p < 0.0001)* | *Age (1: 74 years or younger, 2: 75～ 84 years, 3: 85 years or older):*  Regression coefficient= -0.148  Standard error=0.101  Standardised regression coefficient= -0.112  P-value=0.1481  *Gender (1: Female, 2: Male):*  Regression coefficient= 0.114  Standard error=0.129  Standardised regression coefficient=0.066  P-value=0.3802  *Lower extremity varicose veins (1: none, 2: grade 1, 3: grade 2):*  Regression coefficient=0.272  Standard error=0.108  Standardised regression coefficient=0.188  P-value=0.0132  *Low albuminemia (1: absent, 2: present):*  Regression coefficient=0.704  Standard error=0.209  Standardised regression coefficient=0.244  P-value=0.0010 | *Heart disease (1: none, 2: present):*  Regression coefficient=-0.221  Standard error=0.204  Standardised regression coefficient=-0.084  P-value=0.2810  *Heart failure (1: absent, 2: present):*  Regression coefficient= -0.221  Standard error=0.204  Standardised regression coefficient= -0.084  P-value=0.2810  *Bone and joint disorders (1: none, 2: present):*  Regression coefficient=0.108  Standard error=0.145  Standardised regression coefficient=0.059  P-value=0.4551  *Diabetes (1: None, 2: Present):*  Regression coefficient=0.522  Standard error=0.183  Standardised regression coefficient=0.211  P-value=0.0051  *Renal impairment (1: absent, 2: present):*  Regression coefficient=0.203  Standard error=0.309  Standardised regression coefficient=0.051  P-value=0.5126 | Polypharmacy (1: no, 2: yes):  Regression coefficient= 0.131  Standard error=0.138  Standardised regression coefficient= -0.076  P-value=0.3442  CCB (1: no, 2: yes):  Regression coefficient=0.093  Standard error=0.146  Standardised regression coefficient= 0.052  P-value=0.5258  ARB (1: no, 2: yes):  Regression coefficient=0.068  Standard error=0.147  Standardised regression coefficient= -0.037  P-value=0.6458  *Diuretics (1: None, 2: Present):*  Regression coefficient=0.203  Standard error=0.179  Standardised regression coefficient=0.088  P-value=0.2609 | *Mobility (1: independent walking, 2: walking with a cane, 3: wheelchair use):*  Regression coefficient=0.281  Standard error=0.145  Standardised regression coefficient=0.162  P-value=0.0544  *Daytime activity level (1: active, 2:*  *bedridden, 3: seated):*  Regression coefficient= -0.226  Standard error=0.070  Standardised regression coefficient= -0.244  P-value=0.0016 |
| Kataoka H 2013#  *Backward logistic regression including variables of P<0.02 by univariate analysis.* | Age (≥72y):  OR of leg =2.24 (95%CI 0.97, 5.17). P-value =0.06  Gender:  OR of leg =0.826 (95%CI 0.346, 1.98). P-value =0.67  Varicose veins in leg:  OR of leg =8.18 (95%CI 3.92, 17.1). P-value <0.0001 |  | Use of diuretics:  OR of leg =1.48 (95%CI 0.587, 3.74). P-value =0.41  Treatment for diabetes:  OR of leg =1.72 (95%CI 0.587, 3.74). P-value =0.33 |  |
| O'Connor M et al. 2025*  *Step-wise binary logistic regression* | Gender (female):  OR =1.52 (95%CI 0.96, 2.40)  P-value =0.076 | Chronic heart failure/ ischaemic heart disease:  OR =3.73 (95%CI 2.41, 5.79)  P-value <0.001  Dementia:  OR of leg =0.5 (95%CI 0.35, 1.01). P-value = 0.056 |  | Uses mobility aid:  OR =2.38 (95%CI 1.51,3.75)  P-value <0.001 |

*****Multivariable logistic regression, #Multivariate logistic regression, CI- Confidence interval, n/s not stated, n/a not available for ≥60 years,

ARB Angiotensin II Receptor Blocker, CCB Calcium Channel Blocker, OR Odds ratio, PGZ Pioglitazone, NSAID Non-Steroidal Anti-Inflammatory
